# Disinfection by Heat

**Published:** 1877-11

**Authors:** 


### DISINFECTION BY HEAT.

In a late address, Dr. Seaton, of London, says: "In disinfection by heat there are two objects to accomplish, viz:

1. "To insure the materials being raised throughout to a temperature that can be depended on.

2. "To do this without singeing the goods.

"Penetration takes place much more easily when the article is dry; a horsehair pillow with the normal amount of hygrometic moisture, requires to be heated for eight hours. White wool, linen, cotten, silk and paper may be heated to 250° F., for three hours without apparent injury. A heat of 295° F., continued for three hours, singes white wool, (less so gray), white cotton, white silk, white paper and linen, but does not injure their appearance materially. For penetration of heat into a bed, an exposure of eight hours is required; 250° F. is the best and safest maximum heat to use. Good bed covers, such as are used in hospitals, bear at least six bakings without material change in aspect; a slight and gradually increasing brownise tinge occurs after many bakings, and after a time their appearance and strength are undoubtedly injured; blankets are less changed. The injury caused by periodical stoving of bedding, etc., such as that described above, does not amount to more than what may be called fair wear and tear.—*Med. and Surg. Reporter.*

